# Supplementary material for: Naïve Huntington’s disease microglia mount a normal response to inflammatory stimuli but display a partially impaired development of innate immune tolerance that can be counteracted by ganglioside GM1
Source: J Neuroinflammation. 2023 Nov 23;20:276. doi: 10.1186/s12974-023-02963-y (PMC10668379; doi:10.1186/s12974-023-02963-y)

SUPPLEMENTARY INFORMATION

Naïve Huntington's disease microglia mount a normal response to inflammatory stimuli but display a partially impaired development of innate immune tolerance that can be counteracted by ganglioside GM1.

Noam Steinberg, Danny Galleguillos#, Asifa Zaidi, Melanie Horkey and Simonetta Sipione*

Department of Pharmacology and Neuroscience and Mental Health Institute, University of Alberta Edmonton, AB, Canada

*** Correspondence**
Simonetta Sipione
Email: ssipione@ualberta.ca

\# **Current address**
Department of Neurology, Johns Hopkins University School of Medicine, Baltimore, MD, USA

Keywords: Huntington's disease, Q140/140 knock-in mice, ganglioside, neuroinflammation, LPS, TLR-4, TLR-2, tolerance, GM1, microglia.

SUPPLEMENTARY FIGURE LEGENDS

Supplementary Fig. 1. Q7/7 and Q140/140 microglia express similar levels of TLR4 and TLR2 at the plasma membrane in naïve and stimulated conditions. (A) Schematic representation and timeline of cell treatment with LPS. Representative histograms and relative flow cytometry quantification (% TLR4$^+$-cells and median fluorescence intensity) of plasma membrane TLR4 in naïve microglia (I), after 12 h of exposure to LPS (100 ng/ml) (II), and after LPS removal and 24 h of recovery in serum-free medium (III). N ≥ 5. A two-sided unpaired *t*-test was used to compare TLR4 levels between genotypes. **(B)** Plasma membrane TLR2 was measured by flow cytometry in naïve microglia (I) and after 6 h of LTA (10 ug/ml) stimulation (II). Representative histograms and quantification of TLR2$^+$-cells and TLR2 median fluorescence intensity are shown in the bar graphs. N≥3. Two-way ANOVA with Tukey's multiple comparisons test. Bars are means ± STDEV.

Supplementary Fig. 2. LPS treatment does not significantly affect the survival of Q7/7 and Q140/140 microglia. Representative images of Q140/140 microglia stained with Hoechst (blue) and PI (yellow) after incubation in serum-free medium for 24 h (top panels), and Metaxpress software masks (bottom panels) used for the automated quantification of cell nuclei and propidium iodide (PI)-positive cells (dead cells) by high-content microscopy analysis of cell death. Scale bar = 150 μm. The graph shows the % of PI-positive cells in microglia cultures treated with or without LPS (100 ng/ml) for 24 and 48 h. N=4. Two-way ANOVA with Tukey's post test.

Supplementary Fig.3: Necrotic N2a cells carrying mutant HTT induce higher microglial expression of pro-inflammatory cytokines compared to necrotic cells carrying wild-type HTT. Q7/7 and Q140/140 microglia were incubated with necrotic N2a25Q (25Q, wild-type HTT) or N2a97Q (97Q, mutant HTT) cells for 4 h (1:2 microglia to necrotic cells ratio). Graphs show

the fold-change of pro-inflammatory cytokine gene expression compared to the expression induced by necrotic N2a25Q cells. mRNA levels of the indicated cytokines were normalized over the geometric mean of three housekeeping genes (Normalization Index) ($N \geq 4$). Ratio paired *t*-test. *p<0.05.

Supplementary Fig. 4. Comparable levels of cell death in Q7/7 and Q140/140 microglia after exposure to LPS and recovery. LDH enzymatic activity released in the culture medium due to cell death was measured in microglia cultures incubated with or without LPS (100 ng/ml) for 12 h (**A**) and after 24 h recovery in serum-free medium. N=3. (**B)** Two-way ANOVA with Tukey's multiple comparisons test. Bars are means ± STDEV. *p<0.05.

Supplementary Fig. 5: GM1 decreases expression and production of pro-inflammatory cytokines in Q7/7 microglia. Q7/7 microglia were activated with LPS (100 ng/ml) for 3 h, washed and treated with GM1 (50 µM) for 6 h. GM1 reduced the levels of **(A)** *Il-1b* and *Tnf* mRNA (N=5), and **(B)** TNF secreted in the medium ($N \geq 3$). Gene expression was normalized over *Ppia*. Two-way ANOVA with Tukey's multiple comparisons test was used. **p<0.05; **p<0.01; ***p<0.001, ****p<0.0001.*

Supplementary Fig. 6: GM1 does not affect *Irak-3* expression in naïve Q7/7 and Q14/140 microglia. Naïve microglia were incubated with GM1 in serum-free medium for 8 h prior to RNA extraction and analysis of Irak-3 mRNA levels. Irak-3 expression was normalized over the geometric mean of three housekeeping genes. No statistically significant differences were detected among groups. $N \geq 3$. Bars are means ± STDEV. Two-way ANOVA with Tukey's multiple comparisons test.

Supplementary Fig. 1

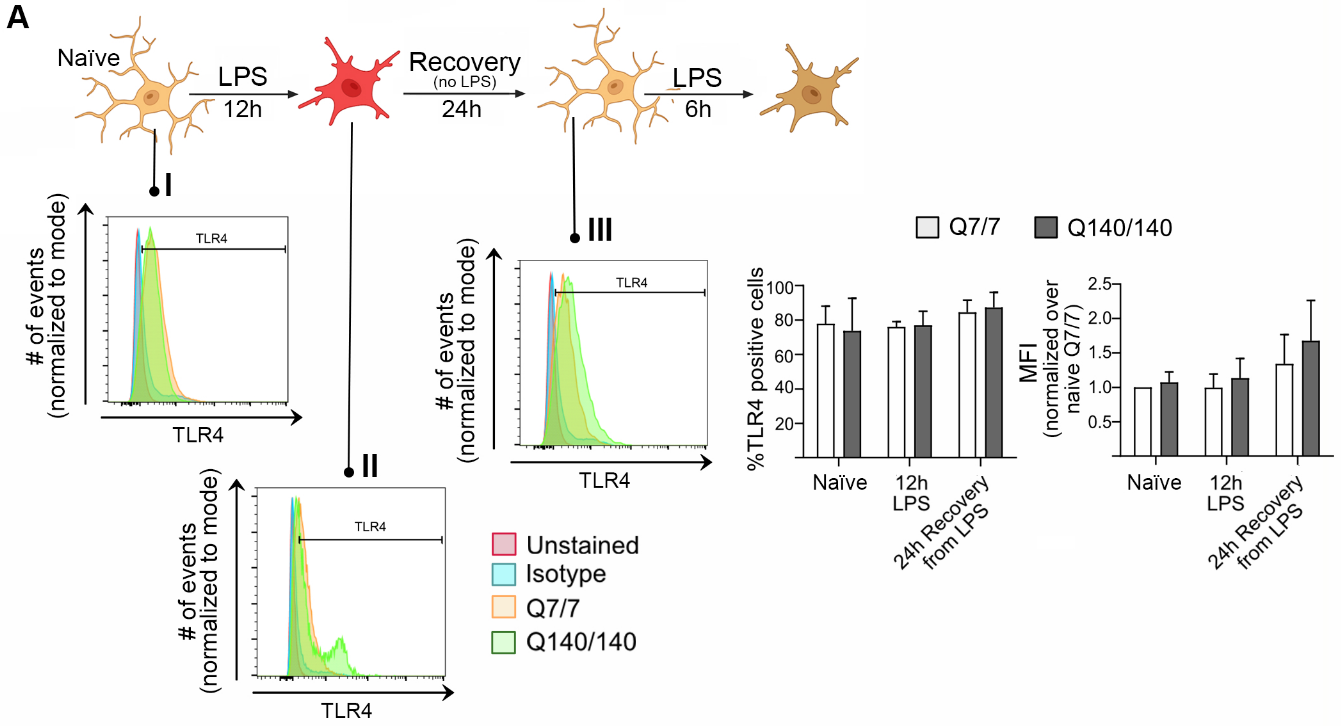

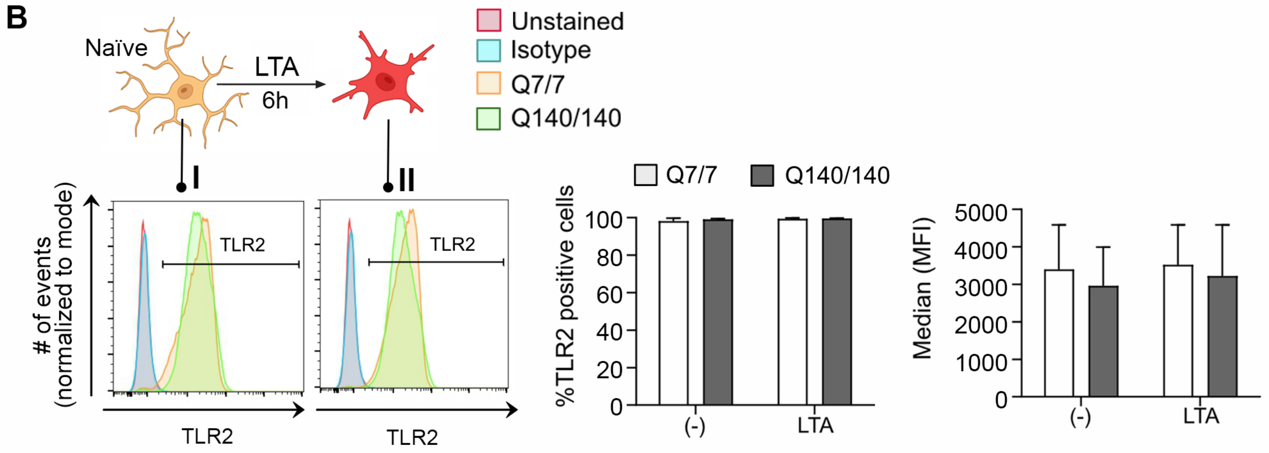

Supplementary Fig. 2

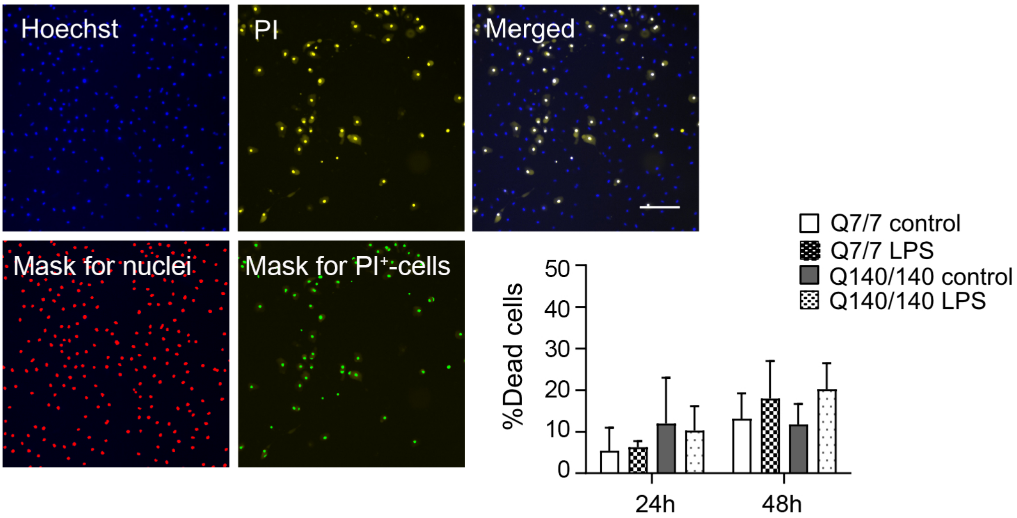

Supplementary Fig. 3

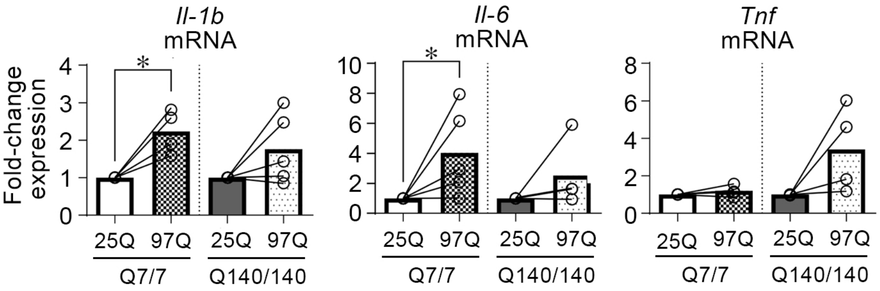

Supplementary Fig. 4

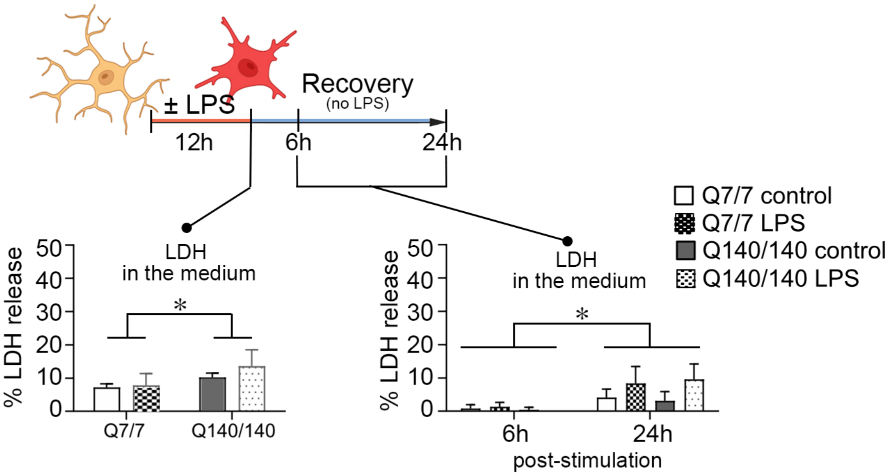

Supplementary Fig. 5

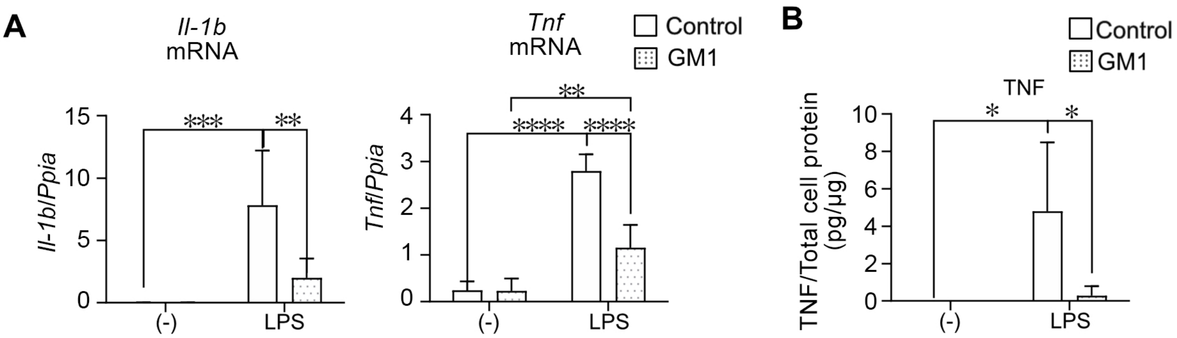

Supplementary Fig. 6

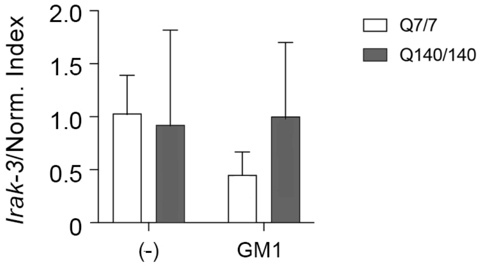

Supplement: Supplementary file 1 — Additional file 1: Fig. S1. Q7/7 and Q140/140 microglia express similar levels of TLR4 and TLR2 at the plasma membrane in naïve and stimulated conditions. (A) Schematic representation and timeline of cell treatment with LPS. Representative histograms and relative flow cytometry quantification (% TLR4+-cells and median fluorescence intensity) of plasma membrane TLR4 in naïve microglia (I), after 12 h of exposure to LPS (100 ng/ml) (II), and after LPS removal and 24 h of recovery in serum-free medium (III). N ≥ 5. A two-sided unpaired t-test was used to compare TLR4 levels between genotypes. (B) Plasma membrane TLR2 was measured by flow cytometry in naïve microglia (I) and after 6 h of LTA (10 ug/ml) stimulation (II). Representative histograms and quantification of TLR2+-cells and TLR2 median fluorescence intensity are shown in the bar graphs. N ≥ 3. Two-way ANOVA with Tukey’s multiple comparisons test. Bars are means ± STDEV. Fig. S2. LPS treatment does not significantly affect the survival of Q7/7 and Q140/140 microglia. Representative images of Q140/140 microglia stained with Hoechst (blue) and PI (yellow) after incubation in serum-free medium for 24 h (top panels), and Metaxpress software masks (bottom panels) used for the automated quantification of cell nuclei and propidium iodide (PI)-positive cells (dead cells) by high-content microscopy analysis of cell death. Scale bar = 150 μm. The graph shows the % of PI-positive cells in microglia cultures treated with or without LPS (100 ng/ml) for 24 and 48 h. N = 4. Two-way ANOVA with Tukey’s post test. Fig. S3. Necrotic N2a cells carrying mutant HTT induce higher microglial expression of pro-inflammatory cytokines compared to necrotic cells carrying wild-type HTT. Q7/7 and Q140/140 microglia were incubated with necrotic N2a25Q (25Q, wild-type HTT) or N2a97Q (97Q, mutant HTT) cells for 4 h (1:2 microglia to necrotic cells ratio). Graphs show the fold-change of pro-inflammatory cytokine gene expression compared to the e [file 12974_2023_2963_MOESM1_ESM.pdf]
